# Ablation Index‐guided point‐by‐point ablation versus Grid annotation‐guided dragging for pulmonary vein isolation: A randomized controlled trial

**DOI:** 10.1111/jce.15294

**Published:** 2021-11-24

**Authors:** Mark J. Mulder, Michiel J. B. Kemme, Luuk H. G. A. Hopman, Amaya M. D. Hagen, Peter M. van de Ven, Herbert A. Hauer, Giovanni J. M. Tahapary, Albert C. van Rossum, Cornelis P. Allaart

**Affiliations:** ^1^ Department of Cardiology, Amsterdam UMC, Vrije Universiteit Amsterdam Amsterdam Cardiovascular Sciences Amsterdam The Netherlands; ^2^ Department of Epidemiology and Data Science, Amsterdam UMC Vrije Universiteit Amsterdam Amsterdam The Netherlands; ^3^ Cardiology Centers of the Netherlands Amsterdam The Netherlands; ^4^ Department of Cardiology North West Clinics Alkmaar The Netherlands

**Keywords:** Ablation Index, atrial fibrillation, catheter ablation, Grid annotation, pulmonary vein isolation

## Abstract

**Introduction:**

Radiofrequency (RF) atrial fibrillation (AF) ablation using a catheter dragging technique may shorten procedural duration and improve durability of pulmonary vein isolation (PVI) by creating uninterrupted linear ablation lesions. We compared a novel AF ablation approach guided by Grid annotation allowing for “drag lesions” with a standard point‐by‐point ablation approach in a single‐center randomized study.

**Methods:**

Eighty‐eight paroxysmal or persistent AF patients were randomized 1:1 to undergo RF‐PVI with either a catheter dragging ablation technique guided by Grid annotation or point‐by‐point ablation guided by Ablation Index (AI) annotation. In the Grid annotation arm, ablation was visualized using 1 mm³ grid points coloring red after meeting predefined stability and contact force criteria. In the AI annotation arm, ablation lesions were created in a point‐by‐point fashion with AI target values set at 380 and 500 for posterior/inferior and anterior/roof segments, respectively. Patients were followed up for 12 months after PVI using ECGs, 24‐h Holter monitoring and a mobile‐based one‐lead ECG device.

**Results:**

Procedure time was not different between the two randomization arms (Grid annotation 71 ± 19 min, AI annotation 72 ± 26 min, *p* = .765). RF time was significantly longer in the Grid annotation arm compared with the AI annotation arm (49 ± 8 min vs. 37 ± 8 min, respectively, *p* < .001). Atrial tachyarrhythmia recurrence was documented in 10 patients (23%) in the Grid annotation arm compared with 19 patients (42%) in the AI annotation arm with time to recurrence not reaching statistical significance (*p* = .074).

**Conclusions:**

This study shows that a Grid annotation‐guided dragging approach provides an alternative to point‐by‐point RF‐PVI using AI annotation.

## 1 INTRODUCTION

Pulmonary vein isolation (PVI) using radiofrequency (RF) energy is an important treatment option in symptomatic atrial fibrillation (AF) patients.^1^ The incidence of AF recurrence following PVI is substantial and can largely be attributed to reconnection of pulmonary veins (PVs) after initial isolation.^2^ To improve RF lesion transmurality and PVI durability, Ablation Index (AI; Carto3, Biosense Webster, Diamond Bar) was developed as a marker of ablation lesion depth.^3^ AI combines contact force, ablation duration, and ablation power into a weighted formula and can be used to provide real‐time information on lesion maturation during ablation procedures. Findings from a recent meta‐analysis suggest that AI guidance during PVI may improve long‐term efficacy while maintaining a favorable safety profile.^4^ However, a limitation of ablation strategies guided by AI is the impossibility to use a catheter dragging technique instead of a point‐by‐point ablation approach. Although comparative studies are sparse, ablation using a catheter dragging technique may shorten procedural duration and improve PVI durability by creating uninterrupted linear ablation lesions.^5^, ^6^, ^7^ In addition, catheter displacements up to 3 mm are allowed during AI‐guided ablation when standard catheter stability settings are used. As such, target AI values may be reached despite significant spatial catheter instability. The Carto3 electrophysiology mapping system allows visualization of the precise site of ablation using 1 mm³ grid points projected on the three‐dimensional electroanatomical map of the left atrium (LA). Coloring of these grid points occurs when pre‐specified time and catheter stability criteria are met. Visualization of ablation using this grid (Grid annotation) may provide valuable information on lesion depth, lesion contiguity, and catheter stability, and allows for ablation using the catheter dragging technique. We conducted a single‐center randomized study to compare an ablation approach guided by Grid annotation with a point‐by‐point AI annotation approach during AF ablation.

## 2 METHODS

### 2.1 Study design

This study was a single‐center, randomized, single‐blind trial. Between September 2018 and September 2019, 88 patients referred for catheter ablation of paroxysmal or persistent AF were included. Exclusion criteria were a previous left atrial ablation or surgery procedure, untreated hyperthyroidism, and uncontrolled hypertension defined as systolic blood pressure ≥ 160 mmHg. The study protocol was approved by the local ethics committee and informed consent was obtained from all participants before enrollment. The study was conducted according to the principles outlined in the Declaration of Helsinki and was registered in the Netherlands Trial Registry (NTR7361).

**Figure 1:**
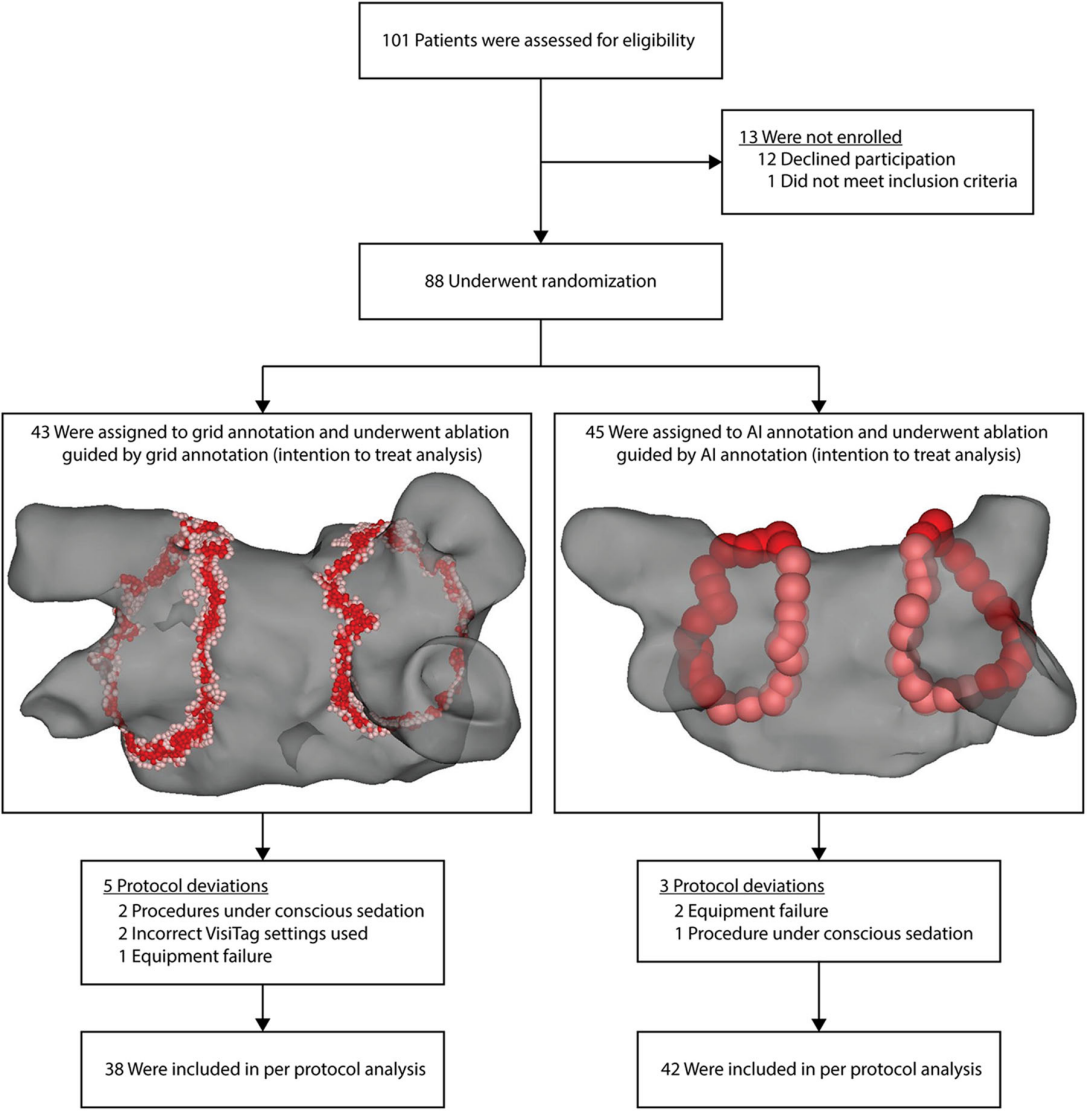
Trial flow chart Ablation Index (AI). Equipment failure included a fatal error of the Carto3 system requiring a restart of the system in two patients (one in Grid annotation arm, one in AI annotation arm) and a defective Patch unit requiring replacement in one patient in the AI annotation arm

After providing written informed consent, patients were randomly assigned in a 1:1 ratio to undergo RF‐PVI either guided by Grid annotation or by AI annotation (Figure 1). The primary endpoint for the study was procedure duration (i.e., time from first RF lesion to end of final left atrial RF lesion).

### 2.2 Ablation procedure

The study protocol mandated that all ablation procedures were performed under deep sedation or general anesthesia. All procedures were performed by three operators (C.A., M.K., and G.T.) with experience of >250 AF RF ablation cases. The modified Seldinger technique was used to obtain femoral access and subsequently one 8F sheath and two 8.5F long sheaths were introduced. Following transseptal puncture, LA geometry was reconstructed using the three‐dimensional fast anatomical mapping algorithm of the CARTO3 electrophysiology system (Biosense Webster, Inc.) with integration of a segmented computed tomography (CT) or cardiac magnetic resonance (CMR) imaging scan. Subsequently, circumferential antral ablation was performed using an irrigated tip contact force (CF)‐sensing ablation catheter (Thermocool Smarttouch, Biosense Webster) to create contiguous lesion circles enclosing ipsilateral PVs. The VisiTag module (Biosense Webster) integrated in the CARTO3 electrophysiology system was used to visualize ablation lesions during ablation. Ablation power was set at 40 W for anterior/roof segments and at 30/35 W for posterior/inferior segments. Although VisiTag settings were used allowing ablation with a minimum CF of 5 g, operators were instructed to pursue a CF between 10 and 40 g during ablation, irrespective of randomization arm. Touch‐up RF applications after initial encircling of the PVs were performed if required to achieve PVI. After a waiting period of 30 min, durability of PVI was assessed without adenosine testing. If acute reconnection occurred, further ablation was performed until re‐isolation was achieved, followed by a new waiting period of 30 min. The ablation strategy was strictly PVI for both randomization arms. Additional ablation lines were not allowed, with the exception of cavotricuspid ablation in case of documented typical counterclockwise atrial flutter.

### 2.3 Grid annotation versus AI annotation

VisiTag settings in the Grid annotation randomization arm were chosen based on consensus of the three operators in the study (MK, GT, and CA) after a learning period in which each operator performed at least 10 procedures with grid visualization of ablation lesions. VisiTag settings were adjusted aiming to balance the risks of both under‐ and overshooting of RF energy. The following VisiTag settings were used in the Grid annotation arm: respiration adjustment “on,” stability maximum range 2 mm, stability minimum time 10 s and force over time set at 50% with a minimum force of 5 g. Ablation was visualized using 1 mm³ grid points projected on the electroanatomic map (Figure 1), with grid coloring set at 10–15 s. As such, grid points colored to red after 15 s of ablation on the exact grid point location meeting the aforementioned VisiTag criteria. Ablation was performed using a dragging technique, aiming for a continuous circle of red grid points.

In the Ablation Index annotation, randomization arm VisiTag settings were used similar to those described by Hussein et al.^8^: respiration adjustment “on,” stability maximum range 3 mm, stability minimum time 8 s and force over time set at 30% with a minimum force of 5 g. Lesion tags were visualized with a diameter of 3 mm and AI target values were set at 380 and 500 for posterior/inferior and anterior/roof segments, respectively. Ablation lesions were created in a point‐by‐point fashion, aiming for a maximum interlesion distance of 6 mm.

### 2.4 Follow‐up and study endpoints

All study participants were followed up for 12 months after PVI and underwent mandatory ECG recordings at 1, 3, 6, and 12 months follow‐up. Twenty‐four‐hour Holter monitoring was performed at 3 and 12 months follow‐up. Furthermore, patients were provided with a mobile‐based one‐lead ECG device (AliveCor Kardia, AliveCor Inc.) and were instructed to use the device when symptoms suggestive of an arrhythmia occurred. Antiarrhythmic drugs were stopped before the end of the blanking period.

The primary endpoint was procedure duration, which was defined as time from first ablation lesion to end of final ablation lesion. Final ablation lesion may be either final lesion of initial PV encircling, final additional touch‐up lesion required to achieve PVI or final lesion to achieve re‐isolation in case of PV reconnection during the procedure. Secondary study endpoints were: (1) proportion of ipsilateral PVs isolated after initial circumferential ablation (first‐pass isolation); (2) proportion of ipsilateral PVs demonstrating acute reconnection during a minimum waiting period of 30 min; (3) freedom from documented atrial tachyarrhythmias after the 90‐day postablation blanking period; and (4) change in patient‐reported symptoms and quality‐of‐life during follow‐up as assessed by disease‐specific AFSS score (Toronto Atrial Fibrillation Severity Scale) and generic SF‐36 score (Short Form Health Survey).

### 2.5 Statistical analysis

Sample size calculation was based on the assumption of a 16‐min improvement in the geometric mean of the primary endpoint (procedure duration) from 104 min with Grid annotation compared to 88 min with AI annotation. Geometric means were estimated on prior observational data.^9^ It was assumed that the natural logarithm of procedure durations were normally distributed with means 4.64 and 4.48 and standard deviations 0.27 and 0.19. Standard deviations were estimated by diving the range of log‐transformed procedure durations by 4. To achieve a power of 80% using two‐sided testing at a significance level of 5%, 72 patients were required. The final sample size was increased to a total of 88 patients considering an attrition rate of 10% and to account for potential loss in power in case nonparametric tests had to be used.

Continuous variables are expressed as mean ± standard deviation in case of a normal distribution or as median (interquartile range) if not normally distributed. Categorical variables are presented as frequency (percentage). Categorical variables were compared using the chi‐square test and continuous variables using the Student's *t* test or Mann–Whitney *U* test when appropriate. Kaplan–Meier analysis was performed to assess freedom of atrial tachyarrhythmias for patients over time with differences between groups tested using the log‐rank test. Analyses of the primary and secondary endpoints were carried out primarily on all randomized patients (intention‐to‐treat population), and additionally on all patients whom underwent study procedures without protocol violations (per‐protocol population). Changes in patient‐reported symptoms and quality‐of‐life during follow‐up were compared between groups using a linear mixed model to account for within‐patient correlations. SPSS (version 26, IBM Corporation) was used for statistical analyses. Two‐sided *p* < .05 were considered statistically significant.

## 3 RESULTS

Enrollment of patients started on September 4, 2018 and was completed on September 26, 2019. A total of 101 patients referred for AF ablation were assessed for eligibility, of whom 88 were enrolled, randomized, and underwent AF ablation (Figure 1). Mean age of the study participants was 62 ± 9 years, 77% of the participants were male, and 69% of patients had paroxysmal AF before ablation. Detailed baseline characteristics stratified by randomization arm are noted in Table 1. Protocol violations were noted in 8 (9%) patients, whom were excluded for the per‐protocol analysis (Figure 1).

**Table 1:** Baseline characteristics

Characteristic	Grid annotation (*n* = 43)	AI annotation (*n* = 45)
Age (years)	60 ± 10	64 ± 7
Male	32 (74%)	36 (80%)
Body length (cm)	181 ± 10	180 ± 9
Weight (kg)	87.8 ± 12.1	86.4 ± 15.9
Body mass index (kg/m²)	26.8 ± 3.6	26.7 ± 3.9
Paroxysmal AF	28 (65%)	33 (73%)
AF duration (months)	44 ± 46	73 ± 63
Number of failed AAD	1.3 ± 0.6	1.3 ± 0.6
Dilated LA	24 (57%)	25 (56%)
GFR (ml/min/1.73 m²)	68 ± 19	67 ± 11
Congestive heart failure	6 (14%)	8 (18%)
Hypertension	15 (35%)	14 (31%)
Diabetes mellitus	4 (9%)	2 (4%)
History of stroke/TIA	4 (9%)	3 (7%)
CHA2DS2‐VASC score	1.5 ± 1.4	1.5 ± 1.1

*Note*: All values are mean ± SD for continuous variables and number (%) for categorical variables.

Abbreviations: AAD, antiarrhythmic drug; AF, atrial fibrillation; GFR, glomerular filtration rate; LA, left atrium; TIA, transient ischemic attack.

### 3.1 Procedural characteristics

**Figure 2:**
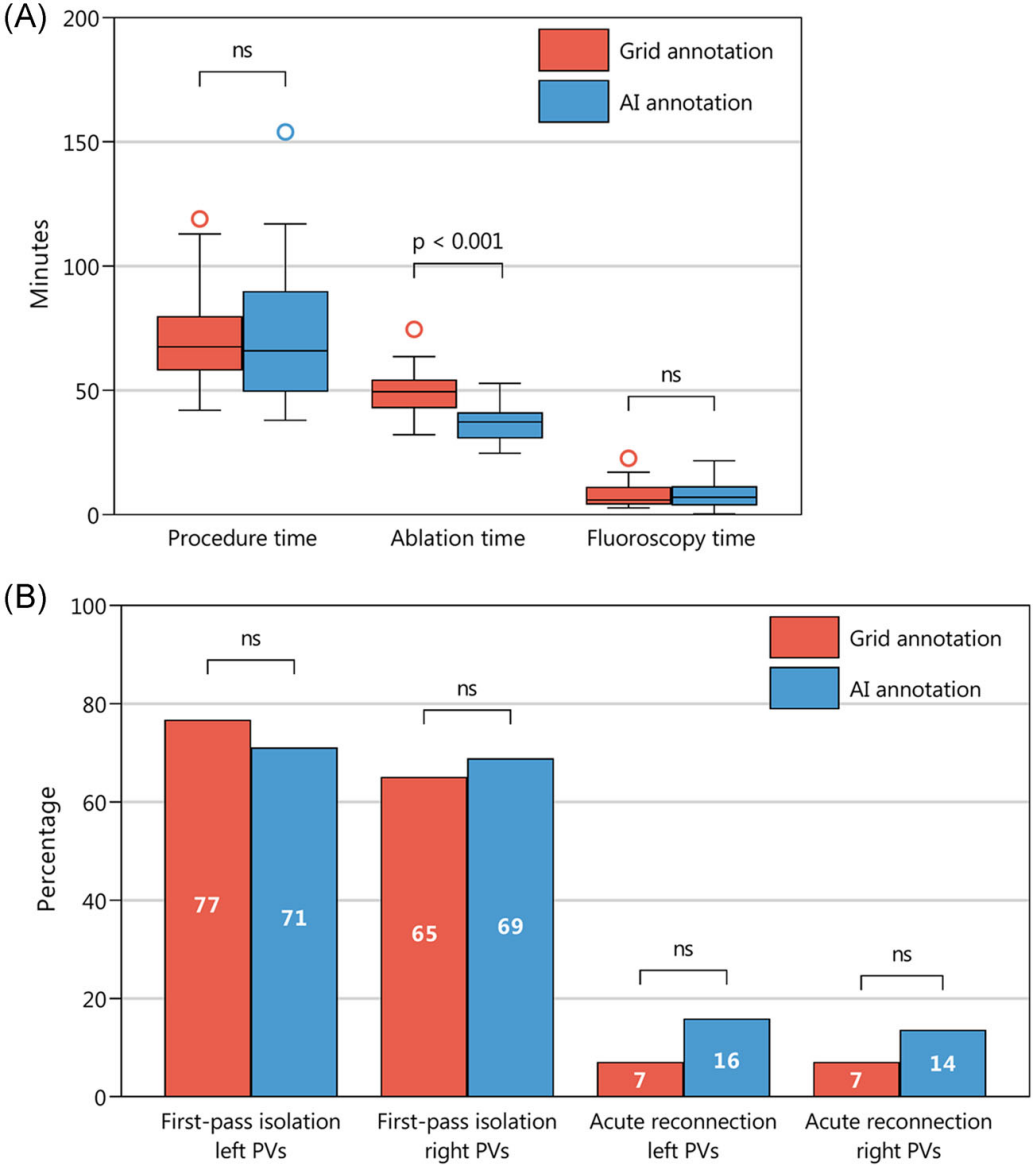
Subgroup assessment of procedural characteristics (intention‐to‐treat). (A) Procedure time (defined as time from first ablation lesion to end of final ablation lesion), ablation time (RF application duration), and fluoroscopy time are shown for both randomization groups by intention‐to‐treat analysis. (B) Percentage first‐pass isolation and acute reconnection for left and right PVs are shown for both randomization groups by intention‐to‐treat analysis. AI, Ablation Index; ns, not significant

In the intention‐to‐treat population, the primary endpoint of procedure duration was not different between the two randomization arms (Grid annotation 71 ± 19 min, AI annotation 72 ± 26 min, *p* = .765, Figure 2). Procedure duration was also not different between the randomization arms in the per‐protocol population (Grid annotation 69 ± 16 min, AI annotation 72 ± 26 min, *p* = .578, Figure S1). RF application time was significantly longer in the Grid annotation arm compared with the AI annotation arm (49 ± 8 min vs. 37 ± 8 min, respectively, *p* < .001). Neither fluoroscopy time nor radiation dose were different between the randomization arms.

The proportion of both left‐sided and right‐sided ipsilateral PVs that were isolated after initial encircling (first‐pass isolation) did not differ between the Grid annotation arm and the AI annotation arm. Similarly, the rate of acute reconnection of left and right PVs were not different between the randomization arms. Table 2 summarizes the procedural characteristics for both the Grid annotation arm and the AI annotation arm.

**Table 2:** Procedural characteristics

Characteristic	Grid annotation (*n* = 43)	Ablation Index annotation (*n* = 45)	*p* value
Procedure duration (min)	71 ± 19	72 ± 26	.765
RF application time (min)	49 ± 8	37 ± 8	**<.001**
Fluoroscopy time (min)	6 [4–11]	7 [4–11]	.983
Radiation dose (Gy x cm²)	64 [47–98]	77 [43–117]	.767
First pass isolation left veins	33 (77%)	32 (71%)	.548
First pass isolation right veins	28 (65%)	31 (69%)	.707
Acute reconnection left veins	3 (7%)	7 (16%)	.205
Acute reconnection right veins	3 (7%)	6 (14%)	.325
Left WACA circumference (mm)	119 ± 18	120 ± 23	.839
Right WACA circumference (mm)	129 ± 18	126 ± 21	.506
CTI ablation	12 (28%)	16 (36%)	.441
Mean LA pressure (mmHg)	10 ± 3	11 ± 5	.254
Mean RA pressure (mmHg)	6 ± 3	6 ± 3	.574

*Note*: All values are mean ± SD or median [IQR] for continuous variables and number (%) for categorical variables.

Abbreviations: CTI, cavo‐tricuspid isthmus; LA, left atrium; RA, right atrium; RF, radiofrequency; WACA, wide area circumferential ablation.

### 3.2 Follow‐up

**Figure 3:**
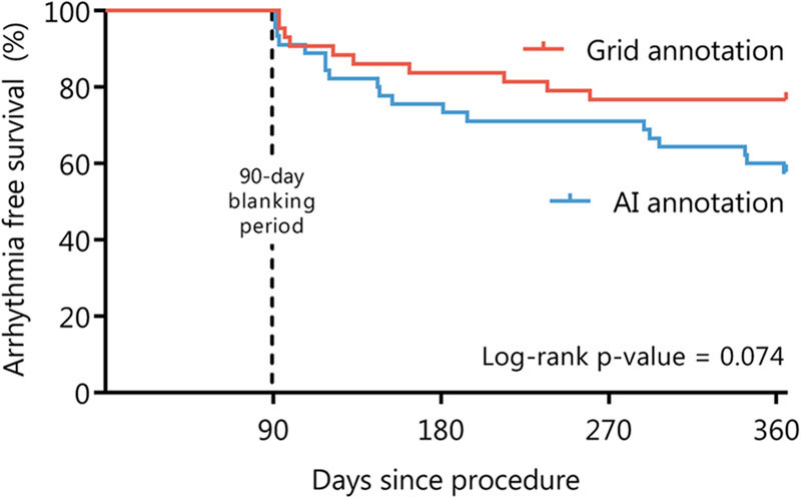
Kaplan–Meier survival analysis for freedom of atrial tachyarrhythmias Kaplan–Meier curves showing freedom from atrial tachyarrhythmias in both randomization arms. AI, Ablation Index

All patients completed 12 months of follow‐up and recurrent atrial tachyarrhythmias were observed in 29 patients (33%). In the intention‐to‐treat population, recurrence of any atrial tachyarrhythmia was documented in 10 patients (23%) in the Grid annotation arm compared with 19 patients (42%) in the AI annotation arm, which did not reach statistical significance by log‐rank test (*p* = .074, Figure 3). In the per‐protocol population, recurrent atrial tachyarrhythmias were observed in eight patients (21%) in the Grid annotation arm compared with 17 patients (40%) in the AI annotation arm (*p* = .077 by log‐rank test, Figure S2).

**Figure 4:**
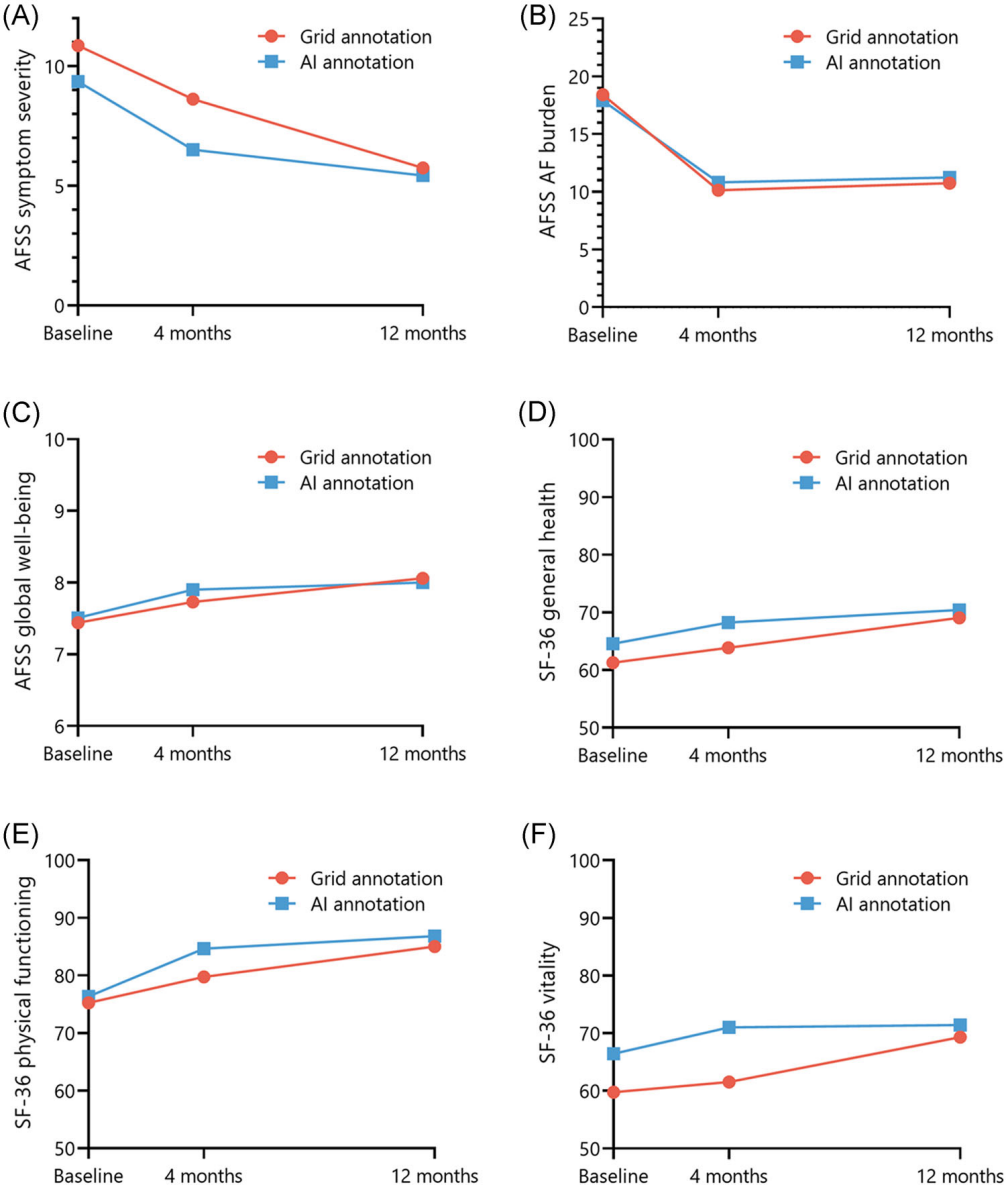
Quality‐of‐life subscores divided by randomization groups Toronto Atrial Fibrillation Severity Scale (AFSS) and 36‐Item Short‐Form Health Survey (SF‐36) quality of life scores at baseline, and 4 and 12 months of follow‐up, divided by randomization group. (A) Mean values of AFSS symptom severity score (lower = better, range: 0–35). (B) Mean values of AFSS AF burden score (lower = better, range: 3–30). (C) Mean values of AFSS global well‐being score (higher = better, range: 1–10). (D) Mean values of SF‐36 general health score (higher = better, range: 0–100). (E) Mean values of SF‐36 physical functioning score (higher = better, range: 0–100). (F): Mean values of SF‐36 vitality score (higher = better, range: 0–100). AI, Ablation Index

All subscales of the disease‐specific AFSS quality‐questionnaire (symptom severity, AF burden, and global well‐being) and generic SFSS questionnaire (general health, physical functioning, vitality) improved between baseline and 12 months of follow‐up (Table S1). There was no statistically significant difference in the improvement of disease‐specific or generic quality‐of‐life scores between the Grid and AI annotation arms (Figure 4).

### 3.3 Complications

Few adverse events occurred in the present study. One postprocedural pericarditis occurred in the Grid annotation arm, which could be managed conservatively. No adverse events occurred in the AI annotation arm (Table S2).

## 4 DISCUSSION

We report on the first randomized study comparing a novel AF ablation approach guided by Grid annotation allowing for “drag lesions” with a standard point‐by‐point ablation approach guided by AI annotation. The main results are as follows: (1) procedural duration did not differ between the two randomization arms; (2) RF application time was significantly longer in the Grid annotation arm; (3) there were 10 patients (23%) in the Grid annotation arm with recurrent AF during follow‐up, compared with 19 patients (42%) in the AI annotation arm, which did not reach statistical significance; and (4) the improvement of disease‐specific or generic quality‐of‐life scores was not different between the randomization arms.

A frequent finding in patients undergoing redo AF ablation after initial successful RF‐PVI is recovery of electrical conduction between the LA and PVs.^10^ Real‐time assessment of ablation using surrogates of RF lesion formation may aid in the creation of reproducible transmural ablation lesions, and may consequently decrease the incidence of PV reconnection and postablation AF recurrence. AI has attracted considerable interest as a guide for PVI procedures and incorporates CF, ablation time, and ablation power into a single value.^3^ AI was shown to correlate strongly with RF lesion size in an animal study and minimum AI values were predictive for PV reconnection after AF ablation.^3^, ^11^ Several studies have investigated the use of AI targets in combination with a maximum interlesion distance to guide AF ablation procedures.^8^, ^12^, ^13^, ^14^, ^15^, ^16^

A recent meta‐analysis of nonrandomized studies found that AI‐guided PVI compared with non‐AI‐guided ablation yielded higher rates of first‐pass isolation, lower rates of acute PV reconnection, and improved atrial tachyarrhythmia survival.^4^ Despite the consistently observed improvement in procedural outcomes, precise ablation strategies differ considerably between studies. Minimal AI targets range from 400 to 600 for anterior/roof segments and from 330 to 450 for posterior/inferior segments, whereas maximum targeted interlesion distance varied between 4 and 6 mm. In this study, we used AI target values of 500 for anterior/roof segments and 380 for posterior/inferior segments, in combination with a maximum interlesion distance of 6 mm. In addition, substantially different catheter stability and CF VisiTag criteria have been used in studies. Maximum distance change ranged between 2.5 and 4 mm for a minimum time ranging from 3 to 15 s, whereas force over time varied between 25% and 60% with a minimum force of 3 to 5 g. Currently, little is known about the biophysical effects of spatial catheter stability on RF lesion formation and the consequences of different criteria have not yet been studied. Future studies are needed to determine the impact of varying stability and CF criteria and to elucidate optimal VisiTag settings.

Previous studies observed recurrent AF during follow‐up in 6%–58% of patients undergoing AI‐guided AF ablation.^4^, ^17^ Our observation of a 42% AF recurrence‐rate is at the high end compared to previous studies. This may be explained by the unfavorable patient characteristics in our unselected patient population, with the majority of patients having a dilated LA and approximately one‐third of patients classified as having persistent AF. Moreover, the strict follow‐up protocol included both mandatory ECGs, Holter‐monitoring and a mobile‐based one‐lead ECG device, maximizing detection of recurrent tachyarrhythmia episodes.^18^ Findings from a recent randomized study suggest that clinical outcomes may also be improved by reducing the maximum target interlesion distance from 6 to 4 mm.^19^

A prerequisite of AI‐guided ablation is the use of a point‐by‐point ablation approach. However, using a catheter dragging technique may be favorable for RF‐PVI by allowing for uninterrupted linear lesions. Furthermore, operators may prefer a dragging catheter ablation technique over point‐by‐point ablation. Ex‐vivo studies have demonstrated that continuous RF applications result in larger ablation lesions than interrupted point‐by‐point lesions.^5^, ^6^ A previous AF ablation study comparing a catheter dragging approach with a point‐by‐point approach found improved procedural time and early AF recurrence rate with catheter dragging ablation, although this study was retrospective in design and did not include CF‐sensing catheters or strategies for objective ablation lesion visualization.^7^

This study introduces a novel Grid annotation ablation approach. This approach instantly visualizes information on catheter movement during ablation (Figure S3), providing direct feedback to the operator on spatial catheter stability. In addition, the automatically projected small grid points provide visual feedback on ablation line continuity. As such, this Grid annotation approach combines the advantages of allowing drag lesions with objective ablation lesion visualization. A limitation of the Grid annotation ablation approach may be that it does not take differences in ablation power settings into account.

Neither arrhythmia recurrence rate, first‐pass isolation rate, nor number of adverse events differed between the randomization arms, although it should be stressed that the study was not powered to detect a difference in these outcomes. Procedure time was anticipated to be shorter in the Grid annotation randomization arm, because of more continuous lines leading to higher number of first pass isolation and a reduced need for repositioning of the ablation catheter before each RF application. Nevertheless, presumably due to the longer RF application time in the Grid annotation arm, procedure time was similar between the two randomization arms. A consequence of longer RF application time may be more transmural and contiguous ablation and therefore more durable lesion sets, but potentially also more complications. The longer RF application time may be explained by the stricter stability and CF criteria in the Grid annotation arm.

### 4.1 Limitations

The most important limitation of the present study is its relatively small sample size, which was not powered to detect a difference in arrhythmia recurrence rate. Furthermore, all procedures were performed by three operators in a single center. Larger multicenter studies are needed to validate the safety and efficacy of the novel Grid annotation ablation approach.

Although ECGs, 24‐h Holter monitoring and a mobile‐based one‐lead ECG device were used for arrhythmia follow‐up, asymptomatic recurrences might have been missed.

Furthermore, AI targets in the AI annotation arm in our study differed slightly from AI targets used in the CLOSE protocol (380 vs. 400 in posterior/inferior segments and 500 vs. 550 in anterior/roof segments),^13^ as these were not yet available during design of this study.

Last, all study procedures were performed using automatic respiratory motion adjustment (ACCURESP, Carto3) applied to the automatic annotation of ablation lesions. A recent study showed that identification of catheter motion may be delayed with use of respiratory motion adjustment, which may lead to inaccurate ablation annotation.^20^ The majority of procedures in this study were performed under deep sedation (with maintenance of spontaneous breathing), which may result in less predictable respiratory movement compared with general anesthesia. Use of general anesthesia may improve catheter stability and ablation annotation, and may improve clinical outcomes.^21^

## 5 CONCLUSIONS

Findings from this first randomized controlled study comparing a catheter dragging approach with a point‐by‐point ablation approach show that Grid annotation provides an alternative to RF‐PVI using AI annotation, allowing for ablation with the catheter dragging technique. Larger, multicenter studies are needed to validate the safety and efficacy of this novel approach.

## Supporting information

Supporting information.
