# Supplementary material for: Ablation Index‐guided point‐by‐point ablation versus Grid annotation‐guided dragging for pulmonary vein isolation: A randomized controlled trial
Source: J Cardiovasc Electrophysiol. 2021 Nov 24;33(1):64–72. doi: 10.1111/jce.15294 (PMC9299027; doi:10.1111/jce.15294)
Supplement: Supplementary file 1 — Supporting information. [file JCE-33-64-s001.docx]

**SUPPLEMENTAL MATERIAL**

**Ablation Index-guided point-by-point ablation versus grid annotation-guided dragging for pulmonary vein isolation: a randomized controlled trial (Optigrid trial)**

Mark J. Mulder, M.D.; Michiel J.B. Kemme, M.D, Ph.D.; Luuk H.G.A. Hopman, M.Sc; Amaya M.D. Hagen, M.D.; Peter M. van de Ven, Ph.D.; Herbert A. Hauer, M.D., Ph.D.; Giovanni J.M. Tahapary, M.D.; Albert C. van Rossum, M.D., Ph.D.; Cornelis P. Allaart, M.D., Ph.D.

**Supplemental figures**

Figure 1. Subgroup assessment of procedural characteristics (per-protocol)

Figure 2. Kaplan-Meier survival analysis for freedom of atrial tachyarrhythmias (per-protocol)

Figure 3. Visualization of spatial catheter stability during ablation with grid annotation

**Supplemental tables**

Table 1. Change in quality-of-life scores for the entire study cohort

Table 2. Safety characteristics


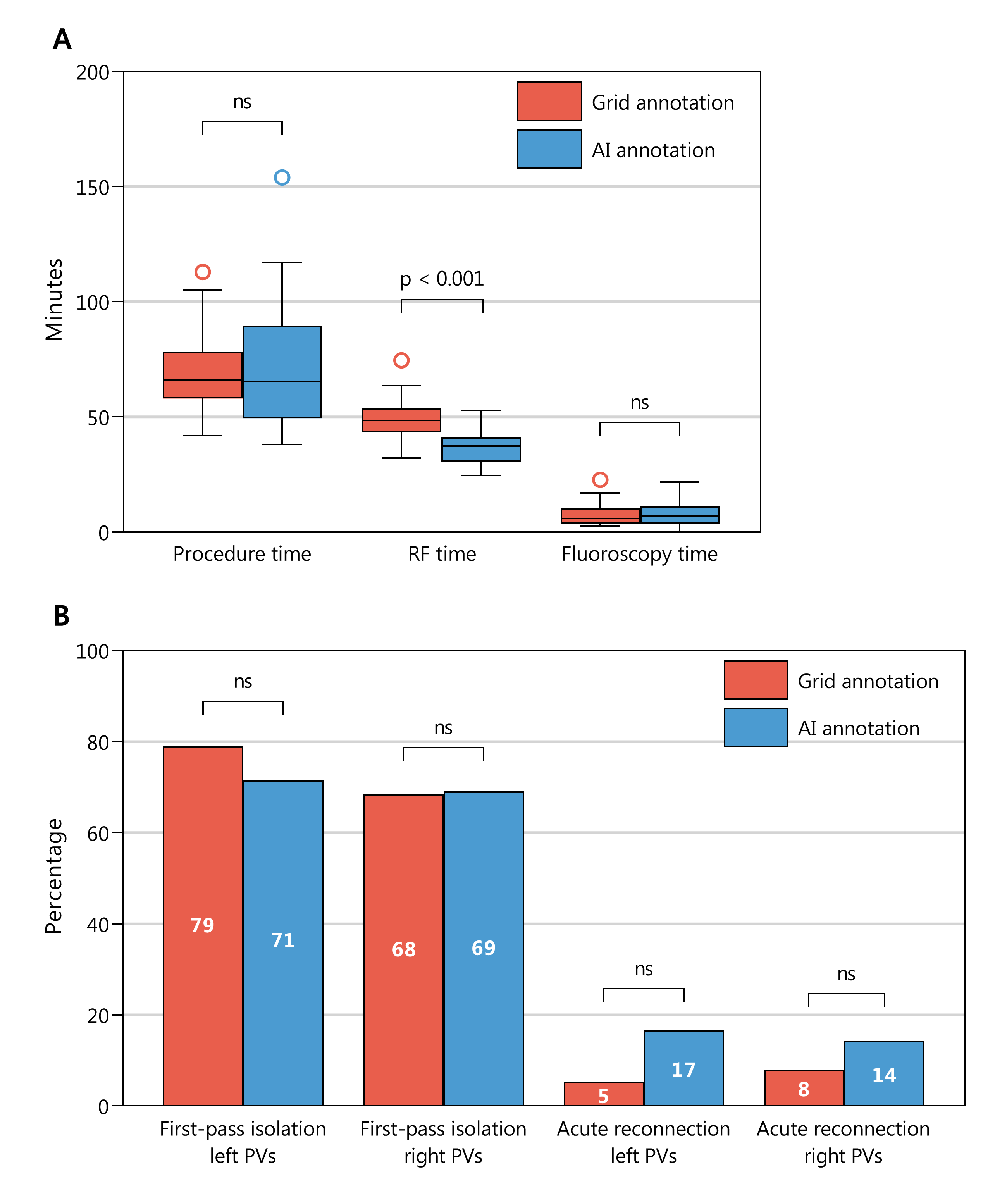


**Supplemental figure 1. Subgroup assessment of procedural characteristics (per-protocol)**

Panel A: Procedure time, ablation time, and fluoroscopy time are shown for both randomization groups by per-protocol analysis. Panel B: Percentage first-pass isolation and acute reconnection for left and right PVs are shown for both randomization groups by per-protocol analysis. AI = Ablation Index, ns = not significant.

**
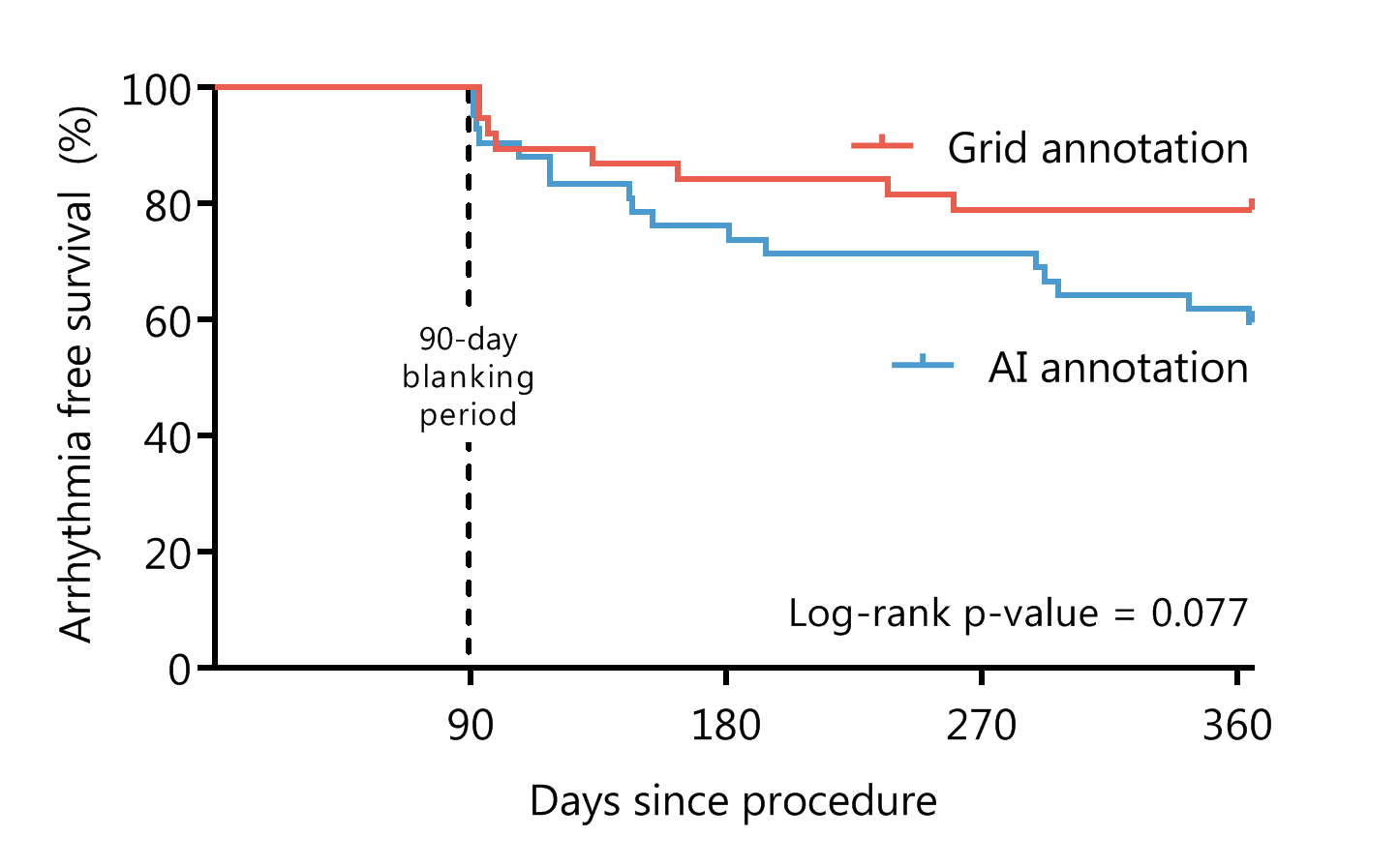
**

**Supplemental figure 2. Kaplan-Meier survival analysis for freedom of atrial tachyarrhythmias (per-protocol)**

Kaplan-Meier curves showing freedom from atrial tachyarrhythmias in both randomization arms of the Optigrid trial by per-protocol analysis. AI = Ablation index.**
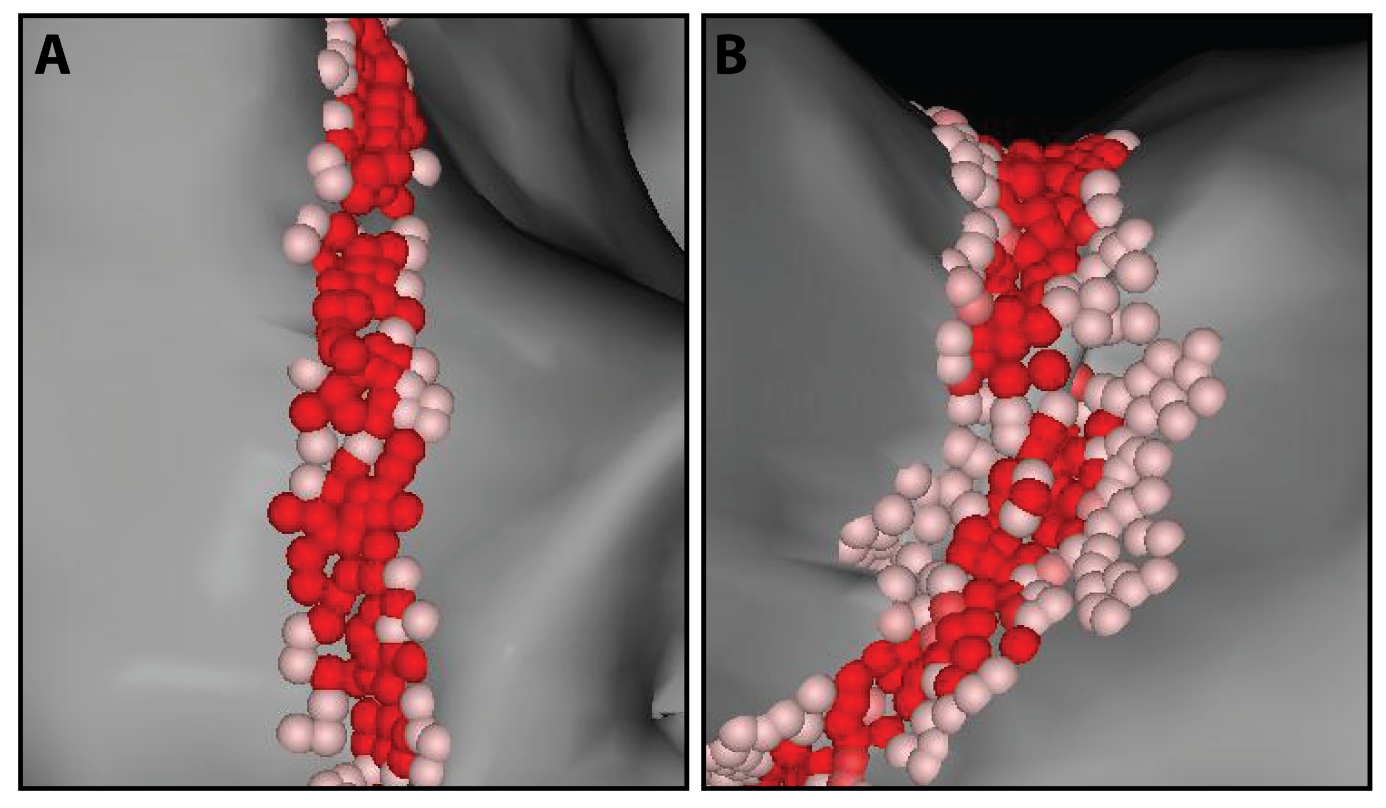
**

**Supplemental figure 3. Visualization of spatial catheter stability during ablation with grid annotation**

Panel A shows an ablation segment, visualized using grid annotation, allowing visualization of the precise site of ablation with 1mm³ grid points projected on the electroanatomic map. This ablation segment shows a narrow rim of grid points, indicating adequate spatial catheter stability during lesion formation. Panel B shows a different ablation segment, also visualized using grid annotation. This ablation segment shows a wide rim of grid points, indicating decreased spatial catheter stability due to catheter movement/sliding.

**Supplemental table 1. Change in quality-of-life scores for the entire study cohort**

| **Quality-of-life score** | **Baseline** | **Follow-up (4 months)** | **Follow-up (12 months)** | **p value baseline vs. 4 months** | **p value baseline vs. 12 months** | **p value 4 months vs. 12 months** |
| --- | --- | --- | --- | --- | --- | --- |
| AFSS symptom severity | 10.1 ± 5.6 | 7.6 ± 6.7 | 5.6 ± 6.1 | **0.002** | **<0.001** | 0.097 |
| AFSS AF burden | 18.1 ± 4.7 | 10.5 ± 5.9 | 10.9 ± 5.7 | **<0.001** | **<0.001** | 1.000 |
| AFSS global well-being | 7.5 ± 1.1 | 7.8 ± 1.4 | 8.0 ± 1.1 | 0.106 | **0.003** | 0.629 |
| SF-36 general health | 63.0 ± 21.8 | 66.1 ± 19.6 | 69.7 ± 20.3 | 0.305 | **0.015** | 0.654 |
| SF-36 physical functioning | 75.8 ± 18.1 | 82.2 ± 19.0 | 85.9 ± 18.8 | **0.001** | **<0.001** | 0.425 |
| SF-36 vitality | 63.2 ± 20.6 | 66.3 ± 19.5 | 70.3 ± 18.6 | 0.501 | **0.006** | 0.238 |

Change in Toronto Atrial Fibrillation Severity Scale (AFSS) and 36-Item Short-Form Health Survey (SF-36) quality of life scores during follow-up quality-of-life scores.

Data expressed as mean ± standard deviation. p values are derived from Bonferroni post-hoc tests. Bold p values indicate statistical significance (p<0.05 with Bonferroni correction for multiple comparisons).

**Supplemental table 2. Safety characteristics**

| **Characteristic** | **Grid annotation (n=43)** | **AI annotation (n=45)** | **p value** |
| --- | --- | --- | --- |
| Death | 0 (0%) | 0 (0%) |  |
| Cerebrovascular accident | 0 (0%) | 0 (0%) |  |
| Transient ischemic attack | 0 (0%) | 0 (0%) |  |
| Atrio-oesophageal fistula | 0 (0%) | 0 (0%) |  |
| Cardiac tamponade | 0 (0%) | 0 (0%) |  |
| Pericarditis | 1 (2%) | 0 (0%) | 0.304 |
| Phrenic paralysis | 0 (0%) | 0 (0%) |  |
| Vascular access complication | 0 (0%) | 0 (0%) |  |

Data expressed as number (percentage). p value derived from chi-squared test.
